# Retraction: Overexpression of SATB1 Is Associated with Biologic Behavior in Human Renal Cell Carcinoma

**DOI:** 10.1371/journal.pone.0301571

**Published:** 2024-04-02

**Authors:** The PLOS ONE Editors

This article [1] was identified as one of a group of articles connected by concerns about image reuse [2–13].

The image concerns in this article [1] affect Figure 4. Specifically,

- The following panels appear similar:
  - Figure 4A Migration 786-O of [1], Fig 4C siMALAT1+miR-inhibitor of [2] when rotated 180°, and Fig 3D Eca-109/pCMV-SOX2 of [3].
  - Figure 4A Migration Control vector of [1] and Fig 3D Eca-109/pCMV-SOX2-Ctr siRNA of [3].
  - Figure 4A Migration SATB1 shRNA of [1], Fig 4C siMALAT1+miR-NC and siMALAT1+miR-429 of [3], and Fig 3D Eca-109/pCMV-SOX2-Slug siRNA of [3].
  - Figure 4A Invasion 786-O of [1], Fig 4D siMALAT1+miR-inhibitor of [2] when rotated 180°, and Fig 3E Eca-109/pCMV-SOX2 of [3].
  - Figure 4A Invasion Control vector of [1] and Fig 3E Eca-109/pCMV-SOX2-Ctr siRNA of [3].
  - Figure 4A Invasion SATB1 shRNA of [1], Fig 4D siMALAT1+miR-NC of [2] when rotated 180°, Fig 4D siMALAT1+miR-429 of [2] when rotated 180°, and Fig 3E Eca-109/pCMV-SOX2-Slug siRNA of [3].
- The Figure 4C Migration Control vector and Invasion Control vector panels appear to partially overlap. In addition, these two panels also appear to partially overlap with the Figure 4C Invasion ACHN panel when flipped along the horizontal axis.

First author CC stated that they were unaware of the panel duplications with [2] and [3]. They provided image files to support Figure 4. The files did not resolve the concerns, and several of the provided images appear to overlap with each other and/or with images reported in [4, 5] as results obtained from a different cell line.

The *PLOS ONE* Editors retract this article [1] due to the above image concerns that call into question the reliability of the reported results.

The authors either did not respond to the retraction decision directly or could not be reached.
